# Can Biological Traits Serve as Predictors for Fishes’ Introductions, Establishment, and Interactions? The Mediterranean Sea as a Case Study

**DOI:** 10.3390/biology11111625

**Published:** 2022-11-07

**Authors:** Paraskevi K. Karachle, Anthi Oikonomou, Maria Pantazi, Konstantinos I. Stergiou, Argyro Zenetos

**Affiliations:** 1 Institute of Marine Biological Resources and Inland Waters, Hellenic Centre for Marine Research, 19013 Attika, Greece; 2 Department of Zoology, School of Biology, Aristotle University of Thessaloniki, U.P.B. 134, 54124 Thessaloniki, Greece

**Keywords:** bio-invasions, fish, neonatives, alien species, Mediterranean, life history traits

## Abstract

**Simple Summary:**

The appearance of a species outside its native area due to human activities (e.g., canal openings, shipping, aquaculture) is described as a biological invasion, and these species are called non-indigenous. The Mediterranean Sea is prone to such an invasion, which is further enhanced by water temperature rising. Moreover, species from the Atlantic further expand their distribution in the Mediterranean, called neonative species, entering through the Gibraltar. Here, we examined the biological traits (e.g., preferred habitat and temperature, length, feeding habits) of fishes present in the Mediterranean Sea, as well as those from the neighboring Atlantic and Red Sea areas, and those that are neonative and non-indigenous in the Mediterranean. We applied state-of-the-art statistical analyses, aiming to describe their biological traits and spot those that could serve as predictors of species that could become neonative and non-indigenous for the Mediterranean. Overall, the results presented here could serve as a baseline for future research, and provide us with a useful tool to plan in advance measures for the protection of native Mediterranean fishes from such invasions.

**Abstract:**

The Mediterranean Sea (MED) is prone to species’ introductions, induced by human activities and/or climate change. Recent studies focus on the biological traits that result in such introductions, yet on a single-area-type approach. Here, we used, analyzed, and compared biological traits derived from FishBase for MED, non-indigenous (NIS) and neonative (NEO) in the Mediterranean, and adjacent Atlantic (ATL) and Red Sea (RS) species. A quantitative trait-based analysis was performed using random forest to determine the importance of traits in the successful establishment in the Mediterranean. MED fishes were mainly demersal, slow growing and small-medium sized, preferring intermediate temperatures. Conversely, ATL were mainly deep-dwelling species, preferring low temperatures. RS and NIS were predominantly reef-associated, thermophilus, and stenothermic. NEO species were stenothermic with preference to intermediate-high temperatures. Omnivores with preference to animals was the most common trophic group among regions. MED species exhibited higher phylogenetic uniqueness (PD_50_) compared to RS and NIS, indicating that they have long ancestral branches and few descendants. Preferred temperature, habitat type preference and maximum reported length (L_max_) and infinite length (L_inf_) were the most important predictors in the establishment process. Overall, the results presented here could serve as a baseline for future research, especially by using more refined and/or additional biological trail estimates.

## 1. Introduction

Invasion by non-indigenous species (NIS) is a common biological phenomenon occurring at different time scales (e.g., [1]) and at all latitudes (e.g., [2]). NIS represent a potential threat to local biodiversity, as well as ecosystem functioning and services [3,4,5]. Recent introductions in the Mediterranean Sea of non-native species is a rather interesting phenomenon not only due to the enclosed nature of the sea but also because introductions are induced by both natural factors (i.e., range expansion facilitated by climate change) through the Gibraltar Strait (neonative species, sensu [6]) and by anthropogenic factors. The latter (also called aliens, allochthonous, exotic), are documented to have entered the Mediterranean basin by a variety of pathways/vectors (i.e., through the Suez Canal (Lessepsian migrants, sensu [7]), ship transferred (ballast and hulls, [8]), aquaculture accidental entries and aquarium releases [9]). The alien Mediterranean fishes have been treated elegantly in a thorough, colorful book published by the Commission Internationale pour l’ Exploration Scientifique de la mer Méditerranée (CIESM) and entitled “CIESM Atlas of exotic species in the Mediterranean—Vol. 1 Fishes” [10].

A key aspect for understanding and managing invasions is species’ traits, especially those which shape their competitive ability by providing a competitive advantage [11], but also the links between traits and successful establishment [12]. Moreover, in recent years, the implementation of horizon scanning in order to foresee future potential NIS has gained ground and has been widely performed (e.g., [13,14,15,16,17]). Key elements in these horizon-scanning exercises are biological traits of the species scored. These traits work as forecasting features, useful for the assessment of establishment, spreading, and potential impacts of NIS on biodiversity and ecosystem services. Moreover, biological traits are required when modeling the distribution patterns of NIS under different climatic scenarios.

This trait-related invasion has been addressed in a number of recent works (e.g., [18,19,20,21]) and has also been tested in range expansion within the invaded regions (e.g., [22,23]). Yet, these efforts have addressed and analyzed the ichthyofauna of one area/regional sea, while there is little comparison with the biota of neighboring areas. In this work, we compared the biological traits of the fish species native to the Mediterranean with those found in the neighboring Red Sea and Atlantic Sea, as well as those that are NIS and neonative (NEO) in the Mediterranean. The following questions were addressed: (a) are there any significant differences in the biological traits of species between the different groups; (b) do introduced species (i.e., NEO and NIS) occupy different niches in the Mediterranean as a result of differences in their biological traits; and (c) which traits could provide a competitive advantage to future invaders? Answers to these questions could potentially act as a powerful tool in horizon scanning, early detection, and warning of future invasions.

## 2. Materials and Methods

### 2.1. Compilation of the Data Set

Catalogs of species were compiled as follows: (a) Mediterranean natives (MED)—based on the information included in Coll et al. [24] and cross-checked with FishBase [25]; (b) Red Sea species (RS) (i.e., species recorded in Red Sea, Egypt) that have not been recorded in the Mediterranean—according to Akel and Karachle [26]; (c) neighboring Atlantic species (ATL) (i.e., ichthyofauna of West Spain, Portugal, and Morocco) not found in the Mediterranean—using information from FishBase; and (d) non-indigenous (NIS) and neonative (NEO) fish species—reported up to December 2021 [27]. All species were merged in one list; if a Red Sea species has been recorded in the Mediterranean, then it would be included in the NIS list only; similarly, if an ATL has entered the Mediterranean, it would appear in the NEO species’ list. There was only one exception, that of *Diodon hystrix* Linnaeus, 1758, which is included in both NIS and ΝΕO species’ list, as it has entered the Mediterranean through both the Suez Canal (NIS) and Gibraltar (range expanding; ΝΕO).

In addition, the biological traits of the fishes in the above lists were extracted from FishBase. These traits included: (a) maximum reported (L_max_) and the von Bertalanffy [28] infinite length (L_inf_); (b) length (L_m_) and age (t_m_) at maturity; (c) lifespan (longevity) and generation time; (d) trophic level (TROPH) and food consumption (Q/B); (e) growth coefficient K of the von Bertalanffy growth function; (f) mean preferred temperature (mean T); (g) resilience (i.e., ability to withstand exploitation) and vulnerability (i.e., catchability). Based on the preferred temperature values, the temperature range of the species was estimated as the difference between maximum and minimum preferred temperature. Furthermore, with respect to trophic level, species were assigned to functional trophic groups, based on by Stergiou and Karpouzi [29], and Karachle and Stergiou [30], as follows: (a) herbivores (H; TROPH = 2.0–2.1), (b) omnivores with a preference for plants (OV; 2.1 < TROPH < 2.9), (c) omnivores with a preference for animal material (OA; 2.9 < TROPH < 3.7), (d) carnivores with a preference for decapods and fish (CD; 3.7 < TROPH < 4.0), and (e) carnivores with a preference for fish and cephalopods (CC; 4.0 < TROPH). Finally, the phylogenetic diversity index (PD_50_ [31]) and habitat type for each species was also recorded.

### 2.2. Statistical Analyses

Trait data were log-transformed after visual check of data normality with Q-Q (quantile-quantile) plots in order to achieve homoscedasticity. To identify potential relationships between continuous traits, Spearman’s correlation coefficient was used. To correct for multiple comparisons, *p*-values were adjusted using false discovery rate (FDR) [32] and significant correlations at a significance level of a = 0.05 were used for the ecological interpretation. To check for independence and strength of association between the categorical traits among regions- origin (i.e., ATL, MED, NEO, NIS, RS), chi-square test [33] was performed and Cramér’s V [34] was estimated, accordingly. Tables known as contingency, or crosstab tables, were formed for the joint distribution of each pair of the categorical traits and were visualized through balloon plots, where each cell contains a dot whose size reflects the relative magnitude of the corresponding component. To test whether there were significant differences in continuous traits among regions-areas of origin (i.e., ATL, MED, NEO, NIS, RS), pairwise comparisons between group levels controlling for the false discovery rate (FDR) were calculated; a graphical summary of statistically significant relationships is depicted with boxplots. Principal component analysis (PCA) was applied to describe the trait space of groups (i.e., ATL, MED, NEO, NIS, RS) in the two-dimensional space. PCA ordination biplot was conducted based on the correlation matrix for 8 traits (habitat type, resilience levels, L_max_, trophic group, vulnerability, preferred mean temperature, K and life span) after the exclusion of correlated variables.

To identify the traits and determine their relative importance in predicting establishment (response variable) between NIS-RS and NEO-ATL, an ensemble method, random forest was employed [35]. Random forest was preferred to other statistical techniques since it shows suitable performance compared to many other classifiers, it is robust against overfitting and works well with continuous and categorical variables [35]. The random forest algorithm works by aggregating the predictions made by multiple decision trees. Every decision tree in the forest is trained on the bootstrapped dataset (80% of the dataset). Then, we predicted establishment for the holdout species that were used to assess the misclassification rate of the tree (Out-Of-Bag (OOB) 20% of the dataset). The final outcome is the average of the results of all the trees, with the number of decision trees being set to 500 and the number of variables tried at each node to 3. We assessed the model performance by calculating the Receiver Operating Characteristics (ROC) of area under the curve (AUC), as an effective measure of accuracy. The AUC ranges from 0.5 (prediction not better than random) to 1 (perfect prediction) [36]. The higher the AUC, the better the model performance is. AUC values > 0.7 indicate a suitable fit of the model to the data.

All analyses were performed in the R statistical and programming environment [37] using corrplot, factoextra, gplots, DescTools, rcompanion, dplyr, party, ggplot2, randomForest, varImp, and ade4 packages.

## 3. Results

**Figure 1:**
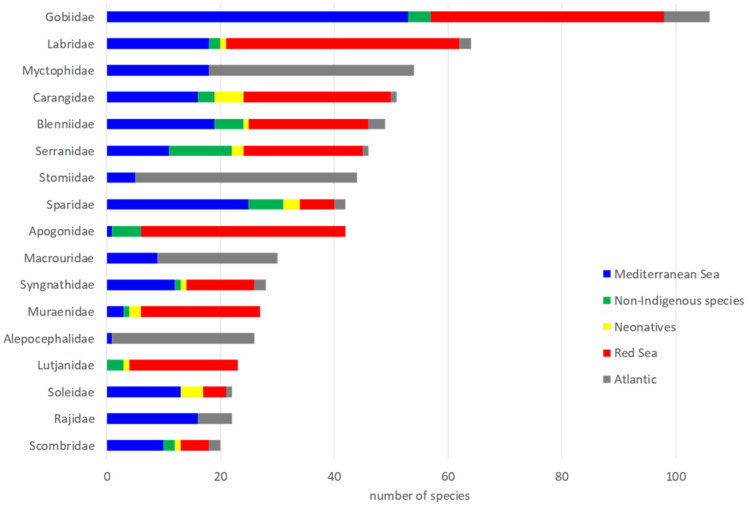
Dominant families (>20 species) in the five different areas examined. Different areas are noted with different colors.

A total of 1673 species belonging to 270 families, was recorded. Gobiidae was by far the dominant family (106 species), followed by Labridae (64 species) and Myctophidae (54 species) (Figure 1), whereas 91 families represented only by one species. Based on the data sources used, 538 species were considered as Mediterranean Sea inhabitants (MED), 517 as Red Sea species (RS), 382 as Atlantic species (ATL), 183 as non-indigenous species (NIS) and 53 as neonative species (NEO), entering from Gibraltar.

### 3.1. Species per Region

#### 3.1.1. Mediterranean Sea Inhabitants (MED)

**Figure 2:**
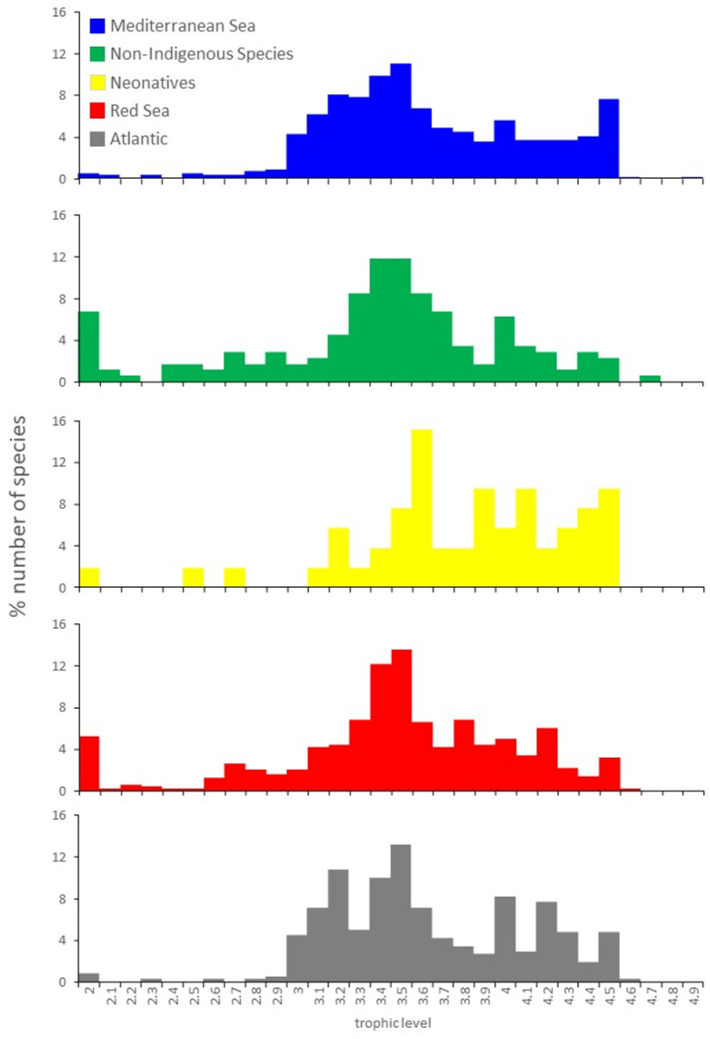
Distribution of species trophic levels per area.

In the Mediterranean basin, Gobiidae (53 species) and Sparidae (25 species) are the dominant out of the 144 families recorded, which is the largest among the areas examined. Almost half of the MED species are demersal (250 species; 46.5%), followed by deep-sea species (19.4%: bathypelagic (11.0%) and bathydemersal species (8.4%)). Reef-associated species were the least represented ones (48 species; 8.9%). Nearly half of the species have medium resilience (245 species; 46.6%) and are of low to moderate vulnerability (356 species; 66.2%) (Table S1). In general, MED species prefer temperatures between 10 and 16 °C (222 species; 44.0%), whereas the number of species preferring low (<6 °C: 20 species, 4.0%) or high (>20 °C: 70 species, 13.9%) temperatures are low (Table S1). More than 75% of the species have a generation time and tm > 5 years and half of the species mature at lengths > 20 cm (Table S2). MED species do not grow fast (K < 0.5 1/year for 68.1% of the species), and reach small to medium sizes (L_inf_ < 40 cm for 286 species, 53.4%) (Figure 2; Table S2). MED species are generally omnivores (Figure 2; Table S2) with a low consumption rate (Q/B < 10 for 68.8% of the species) (Table S2).

#### 3.1.2. Red Sea Species (RS)

In the RS, 96 families have been recorded, with Labridae and Gobiidae being the dominant ones, each represented by 41 species. The ichthyofauna of the RS is dominated by reef-associated (398 species; 76.1%), thermophilus (>26 °C: 454 species, 94.6%), and stenothermic (temperature range < 6 °C: 410 species, 85.6%) species (Table S1). In general, RS species have high resilience (255 species; 55.0%) and low vulnerability (291 species; 56.3%) (Table S1) and small size and mature in young ages (tm < 2 years: 288 species, 58.2%) and small sizes (L_m_ < 15 cm for 251 species, 48.5%) (Table S2). Again, RS species are mostly omnivores (OA: 249 species, 49.4%; Figure 2; Table S2).

#### 3.1.3. Atlantic Species (ATL)

In the Atlantic, the second largest number of families was recorded (129 families). As the majority of the ATL species were deep-dwelling ones (bathydemersal: 72 species, 18.8%; bathypelagic: 194 species, 50.8%), the dominant families were Stomiidae, Myctophidae, Alepocephalidae, and Macrouridae. The prevalence of the deep-sea species parallels the preferred low temperature (<10 °C: 220 species, 61.8%), accompanied by a rather wide range of temperature (0–14 °C for 95.0% of the species) (Table S1). Like their MED counterparts, ATL species also have medium resilience (153 species; 43.6%) and low to moderate vulnerability (286 species; 74.9%) (Figure 2; Table S1). Half of the ATL species (232 species) have a generation time of 2–6 years and a lifespan > 8 years (165 species, 53.1%). For all remaining parameters, t_m_, L_m_, L_inf_ and L_max_ values span over a wide range (Table S2). Nearly all ATL species prey on animal food (TROPH > 3: 373 species; 97.9%) (Figure 2; Table S2).

#### 3.1.4. Non-Indigenous Species (NIS)

Overall, 77 different families were identified in the NIS list, with Serranidae being the dominant one, with 11 species. Reef-associated (111 species; 61.3%), thermophilus (>26 °C: 132 species, 79.0%), and stenothermic (temperature range < 6 °C: 117 species, 69.2%) species were with the highest representation (Table S1). NIS are characterized by medium-high resilience (152 species; 85.9%) and low vulnerability (93 species; 51.4%). They are small-medium sized species with a short generation time (<2 years: 83 species, 48.8%) (Table S2). Sexual maturity is reached early (tm < 3: 126 species; 74.1%) and at small sizes (L_m_ < 20 cm: 101 species; 56.4%) (Table S2). Nearly half of the NIS species are OA, and approximately 20% of them consume plants (herbivores and OV) (Figure 2; Table S2).

#### 3.1.5. Neonative Species (NEO)

NEO species belong to only 38 families, with Carangidae being represented with five species. Species expanding their range from the temperate-tropical Atlantic, display a preference for waters of medium temperature (10–20 °C: 23 species; 44.2%) and rather high temperatures (26–28 °C: 14 species; 26.9%), but within an intermediate temperature range (range 4–10 °C for 67.3% of the species) (Table S1). These species show a medium-low resilience (38 species; 73.1%) and a moderate-high vulnerability (45 species; 84.9%). NEO species have a high longevity being >10 years for 61.5% of the species (Table S2). As in the case of the Atlantic species, the values for other biological parameters (t_m_, L_m_, L_inf_ and L_max_) vary over a (Table S2). The majority of NEO species are carnivores (CD and CC combined: 31 species; 58.5%) (Figure 2; Table S2).

### 3.2. Between Regions Species’ Comparisons

#### 3.2.1. MED and RS

**Figure 3:**
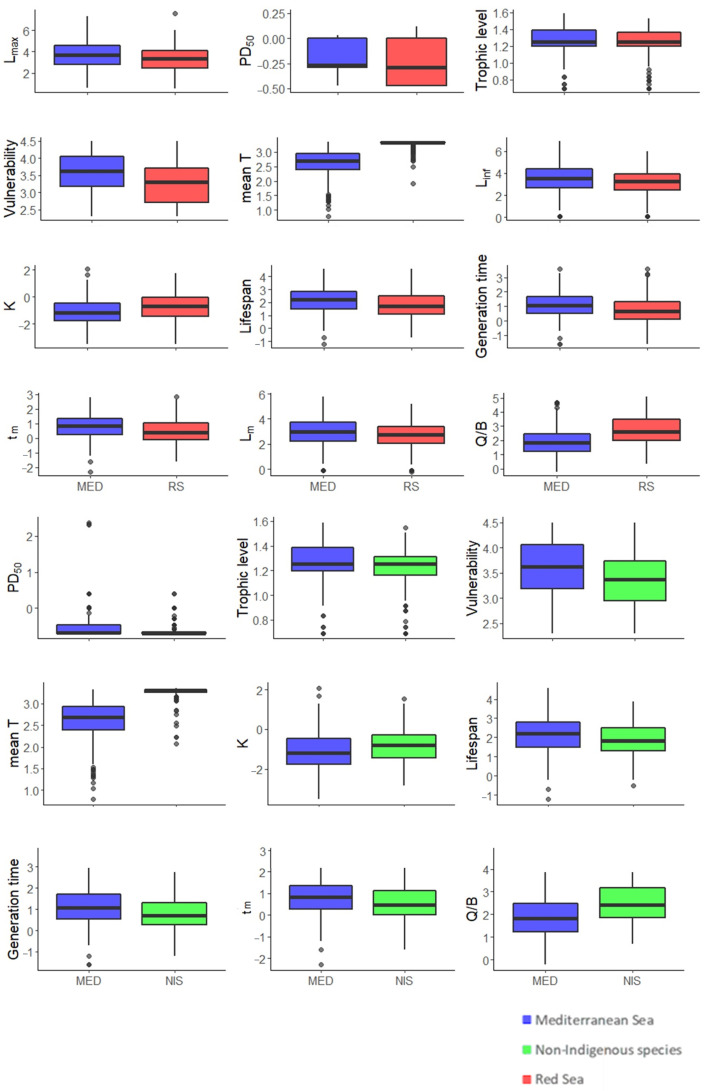
Box-whisker plots of biological traits displaying statistical differences between Mediterranean (MED) (blue) and Red Sea (RS) species (red) (top) and between MED (blue) and non-indigenous (NIS) species (green) (bottom). The central box indicates the spread of values between the 25% and the 75% quartiles, thus representing the 50% of cases around the median (horizontal line); the whiskers (vertical lines) show the range of the values. All variables have been log-transformed. Any point outside the whiskers is identified as an outlier. Mean T: mean preferred temperature; K and L_inf_: von Bertalanffy growth coefficient and infinite length, respectively; L_m_ and t_m_: length and age at maturity, respectively; PD_50_: phylogenetic diversity index; Q/B: food consumption.

All trait comparisons between MED and RS were statistically significant (*p*-value adjusted <0.05), with higher L_max_ (t = 5.5224), PD_50_ (t = 4.8107), TROPH (t = 4.53), vulnerability (t = 8.1427), L_inf_ (t = 4.7035), lifespan (t = 6.6884), generation time (t = 7.5267), t_m_ (t = 7.0304) and L_m_ (t = 4.4346) for the MED fishes. Preferred mean temperature (t = −35.063), K (t = −6.8104) and Q/B (t = −13.309) were lower in the MED than those in the RS (Figure 3).

#### 3.2.2. MED and NIS

MED and NIS differed significantly (*p*-value adjusted <0.05) for PD_50_ (t = 3.4219), TROPH (t = 4.703), vulnerability (t = 4.4327), mean temperature (t = −26.221), K (t = −3.9044), lifespan (t = 3.807), generation time (t = 5.0736), tm (t = 4.4304), and Q/B (t = −7.9175). Of the above-mentioned parameters, mean temperature, K, and Q/B were significantly lower for the MED (Figure 3).

#### 3.2.3. MED and ATL

**Figure 4:**
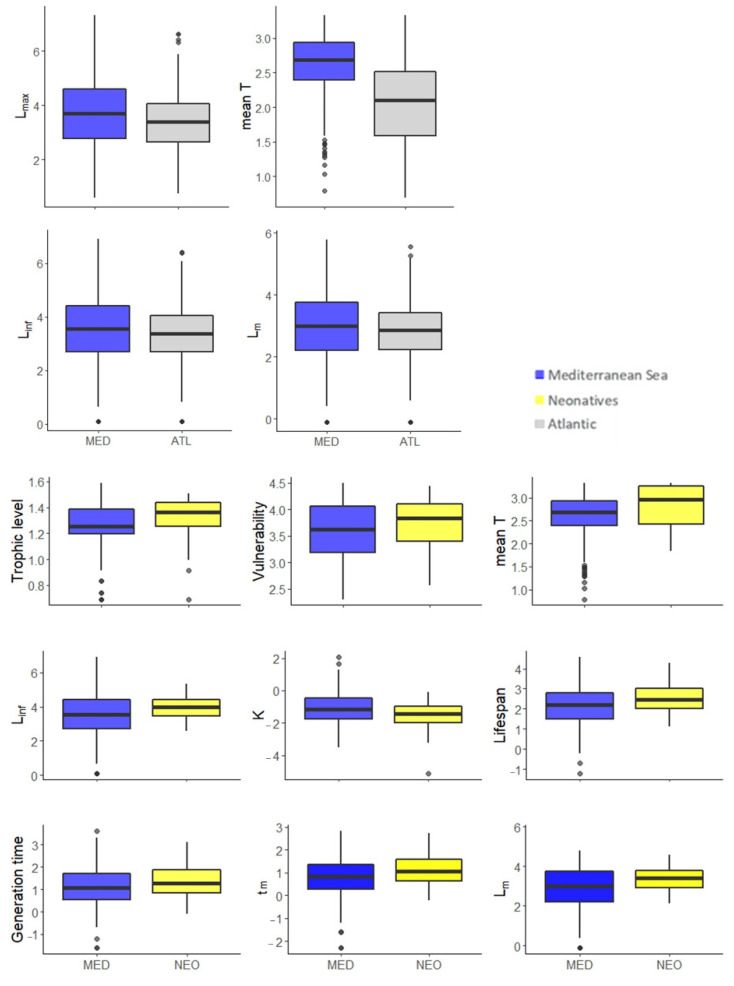
Box-whisker plots of biological traits displaying statistical differences between Mediterranean (MED) (blue) and Atlantic (ATL) species (gray) (top) and between MED (blue) and neonative (NEO) species (yellow) (bottom). The central box indicates the spread of values between the 25% and the 75% quartiles, thus representing the 50% of cases around the median (horizontal line); the whiskers (vertical lines) show the range of the values. All variables have been log-transformed. Any point outside the whiskers is identified as an outlier. Mean T: mean preferred temperature; L_max_: maximum reported length; K and Linf: von Bertalanffy growth coefficient and infinite length, respectively; L_m_ and t_m_: length and age at maturity, respectively.

Only L_max_ (t = 3.64), mean temperature (t = 14.718), L_inf_ (t = 2.4285), and L_m_ (t = 2.2603) differed significantly, being higher for the MED when compared to ATL (*p*-value adjusted < 0.05) (Figure 4).

#### 3.2.4. MED and NEO

MED and NEO species differed significantly (*p*-value adjusted < 0.05), with higher values for MED species being found only for K (t = 3.155). TROPH (t = −1.9468), vulnerability (t = −2.5546), mean preferred temperature (t = −2.9206), L_inf_ (t = −4.1996), lifespan (t = −3.3443), generation time (t = −2.2936), t_m_ (t = −2.7965) and L_m_ (t = −4.4493) were significantly higher for NEO fishes (Figure 4).

### 3.3. Traits per Region

**Figure 5:**
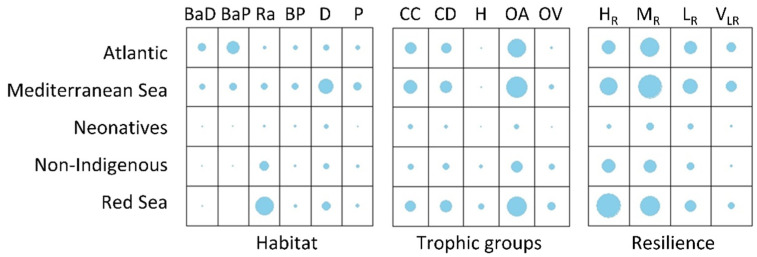
Graphical matrix of the contingency tables formed by levels of region-origin with habitat types (BaD: bathydemersal; BaP: bathypelagic; RA: reef-associated; D: demersal; P: pelagic), trophic groups (H: herbivores; OV: omnivores with a preference for plants; OA: omnivores with a pref-erence for animal material; CD: carnivores with a preference for decapods and fish; CC: carnivores with a preference for fish and cephalopods) and resilience levels (HR: high; MR: medium; LR: low; VLR: very low), where each cell contains a dot whose size reflects the relative magnitude of the corresponding component. ATL: Atlantic; MED: Mediterranean; NEO: neonatives; NIS: non-indigenous, RS: Red Sea.

There was an association between the categorical traits among regions in all cases (Pearson chi-square test of independence, *p*-value < 0.05). Cramér’s V showed that different regions-origin are moderately associated with habitat types (0.43), followed by weak associations of resilience levels (0.16) and different trophic groups (0.13). MED fishes were mainly associated with the demersal habitat type, ATL species with the bathypelagic type, and RS species with the reef-associated one. Omnivores with a preference for animals was the most common trophic group in all regions. Within the RS species, high and medium resilience prevailed, whereas MED and ATL were characterized by lower resilience levels (Figure 5).

The relations between continuous traits indicated 59 statistically significant correlations, incorporating the false discovery rate correction (see Supplementary Material, Figure S1). The von Bertalanffy parameter K had seven strong negative correlations (L_max_, vulnerability, L_inf_, lifespan, generation time, t_m,_ and L_m_), whereas Q/B was also negatively correlated with L_max_, vulnerability, L_inf,_ and L_m_. However, L_max_ was found to have only positive high correlations with vulnerability, generation time, and lifespan. L_max_, L_inf,_ and L_m_ were also positively highly correlated. As expected, L_inf_ and L_m_ were also found to increase with vulnerability, generation time, and lifespan. Vulnerability, generation time, lifespan, and t_m_ were also pairwise highly positively correlated. Finally, in three cases, no strong correlations were detected (mean temperature, PD_50_, TROPH).

### 3.4. Trait Space of Fish Species

The first two principal components of the PCA performed on traits accounted for 74% of the variance among regions. The first axis (PC1, 56.1%) was associated strongly with vulnerability (0.94), lifespan (0.94), K (−0.94), resilience levels (0.86) and L_max_ (0.85), with RS and NIS species showing mainly higher K and low L_max_, vulnerability, lifespan, and resilience. The second principal component (PC2, 17.9%) separated all species from the ATL ones mainly based on habitat type (0.82) and preferred mean temperature (0.80) (Supplementary Material, Figure S2).

### 3.5. Traits of Establishment

**Figure 6:**
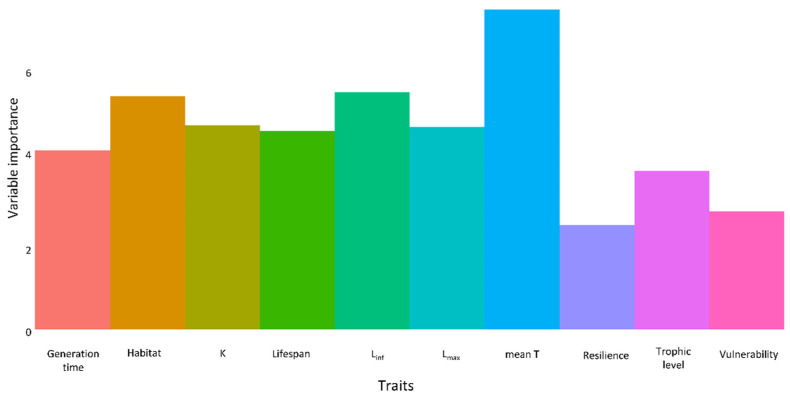
Relative variable importance of traits in predicting establishment between non-indigenous (NIS) and Red Sea (RS) species.

**Figure 7:**
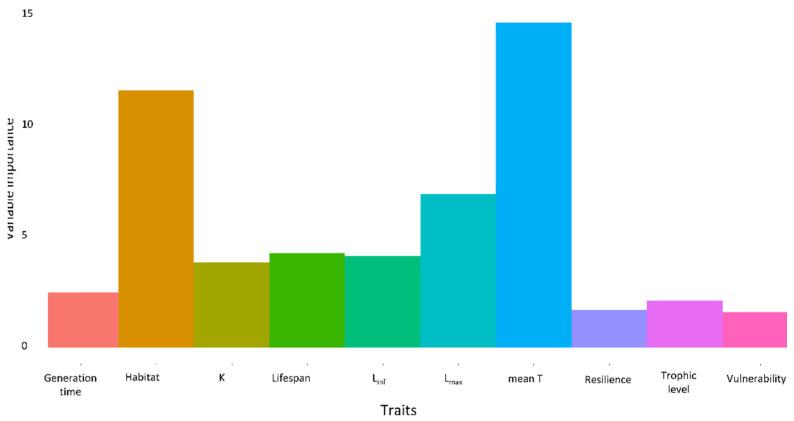
Relative variable importance of traits in predicting establishment between Atlantic (ATL) and neonative (NEO) species.

The variable importance values obtained from the RF analysis between NIS and RRS indicated relatively high importance of three traits—mean T, L_inf,_ and habitat—for predicting establishment (Figure 6), with an AUC value of 0.701 (see Supplementary Material, Figure S3). The variable importance values obtained from the RF analysis between NEO and ATL indicated a relatively high importance of three traits—mean T, L_max,_ and habitat—for predicting establishment (Figure 7), with an AUC value of 0.875 (see Supplementary Material, Figure S4).

## 4. Discussion

### 4.1. Species per Region

The natural opening of Gibraltar has allowed the exchange of biota resulting in an Atlanto-Mediterranean ichthyofauna. The majority of Atlantic fishes, not present in the Mediterranean yet, belong to the deep-sea biota, the biology of which still remains understudied. On the other side of the basin, the opening of the Suez Canal has allowed the entrance of RS species in the Mediterranean, several of which have managed to cross-through the opening, thrive, establish populations, and even become invasive (e.g., *Siganus* spp.; *Fistularia commersonii* Rüppell, 1838; *Pterois miles* (Bennett, 1828) [38]). Yet, there is a number of species that invaded the Mediterranean, presumably introduced by other pathways, such as ship transfer (e.g., *Elates ransonnetti* (Steindachner, 1876) [39]; *Oplegnathus fasciatus* (Temminck and Schlegel, 1844) [40], aquaculture (e.g., *Chanos chanos* (Forsskål, 1775); *Pagrus major* (Temminck and Schlegel, 1843)) and/or aquarium releases (e.g., *Chaetodipterus faber* (Broussonet, 1782) and *Acanthurus cfr gahhm* (Forsskål, 1775) [41]; *Zebrasoma* spp. [9]). Environmental attributes and/or the invading species’ traits could result in a differential successful establishment rate for the two main, geographically distinct, invading origins (i.e., NEO and NIS). NIS, and especially those originating from the Indo-Pacific, are generally highly plastic in terms of eco-physiology [42], growth (with evidence of attaining larger body sizes in the Mediterranean Sea: e.g., [43]), settlement [44], and bathymetric range [45]. This plasticity undoubtedly facilitates their successful establishment in the Mediterranean. In contrast, such information is generally lacking for NEO species. In addition, MED species also exhibit higher PD_50_ values (Phylogenetic uniqueness, index of distinctiveness) compared to RS and NIS species, and no difference with those of the ATL and NEO species. Large PD_50_ values characterize species with long ancestral branches and few other descendants; the Mediterranean phylogenetically distinct species are of relative importance for the potential continuation of evolutionary processes due to the fact that they either lack or have few close taxonomic relatives while they also have relatively distinct genetic diversity [46]. Furthermore, the Mediterranean fish fauna is a suitable example of an assembly of regional faunas through origination and immigration, where dispersal and isolation have shaped the emergence of a biodiversity hotspot [47].

### 4.2. Between Regions Species’ Comparisons

In general, the differences between MED and ATL fishes were few, as opposed to those for all traits between the MED and RS fish fauna were observed. NIS and NEO species, compared to the Mediterranean Sea region, differed in most of the traits studied. Indeed, the interconnection of the Mediterranean and the Atlantic has allowed the free exchange of biota in geological time, and this explains the high affinities between the ATL with those of the Mediterranean. Moreover, the higher values in the mean preferred temperature, all types of lengths, age at maturity, longevity, and generation time of NEO species, compared to the MED ones, indicate that a dynamic potential of biota exchange still exists.

The PCA biplot based on species traits has revealed a high degree of overlap among regions. NIS and RS species demonstrated the smallest trait space, being grouped together, whereas the MED species constituted the center of the trait space. The ATL fish fauna showed the highest trait variation. Principal functional differences among species were associated with vulnerability, lifespan, k, resilience levels, L_max_, habitat type, and preferred temperature. These results are similar to those of Koutsidi et al. [48], that used five life cycle biological traits (longevity, maximum length, trophic level, age at maturity, and fecundity) as important to detect the life history strategies of 205 Mediterranean nektonic species. The RF analysis of species’ traits identified four attributes that are associated with the successful establishment. The mean temperature was the strongest predictor of establishment success, followed by habitat type preference, L_inf,_ and L_max_. These factors probably enhance species establishment likelihood by contributing to their ecological and physiological adaptations and life history adaptations (e.g., [48]). However, the unoccupied functional space occurs when functional distance among native species is high and thus is suitable for the establishment of potential invaders. Invasion success is therefore governed by both the functional traits of non-native species determining their invasiveness and the traits of the invaded community determining how susceptible to invasion it is [49]. It is well known that recent years have witnessed increased functional homogenization (increase in trait similarity) of communities at multiple spatial and temporal scales, with careful attention paid to the species’ abilities to invade new areas (species invasiveness) and the vulnerability of those areas to invasions (community invasibility). Analyzing species invasiveness and community invasibility is expected to have important implications for community and ecosystem properties; thus, it deserves greater attention from marine ecologists (see also [50]).

### 4.3. Biological Traits and Introduction/Establishment

Overall, MED, ATL, and NEO species had higher L_max_, lifespan, vulnerability, and resilience compared to those from the Red Sea and NIS, with the latter displaying higher K values. According to Pauly [51,52] and the gill-oxygen limitation theory (GOLT), high temperatures lower the maximum sizes a fish can attain. The Red Sea is characterized by higher temperatures than those prevailing in the East Mediterranean [53,54], which is showing an increasing trend [55]. The comparisons of traits presented here are in accordance with and strongly support the GOLT theory [51,52], as low L_max_, L_m,_ and longevity, accompanied by a higher growth rate (K coefficient), were lower in the thermophilus RS and NIS species. With respect to vulnerability (i.e., catchability) and resilience (i.e., ability to withstand exploitation), these are two parameters strongly related both to fishing and life history traits. In the case of vulnerability, the estimates of Cheung et al. [56] fuzzy approach are used in FishBase, and hence in the analyses presented here, the higher the fishing pressure, the higher the vulnerability values are. The RS and NIS species were of low vulnerability, combined with high resilience to fishing. The vast majority of these species are reef-associated, occupying a niche that is not heavily fished by large-scale industrial vessels but rather by small-scale artisanal boats, with the major issue for their stocks being climate change (e.g., [57,58,59]). Based on our results and the profile of NEO species (i.e., species that enter the Mediterranean through Gibraltar), the species with the highest likelihood to expand their distribution from the Atlantic in the Mediterranean are demersal and pelagic species that prefer high temperatures. On the other hand, our results showed that RS species that are of high resilience and low vulnerability, combined with high longevity (lifespan), are most likely to enter and establish populations in the Mediterranean.

In all areas examined, omnivores with a preference for animals (OA) prevailed. However, it is noteworthy that in NEO, carnivores with a preference for cephalopods and fishes were equally represented with OA (35.8% and 19 species), whereas carnivores overall (TROPH < 3.7) were more numerous (31 species; 58.4%; Figure 5). In addition, in RS and NIS, herbivores and omnivores with a preference for plants were better represented compared to MED, NEO, and ATL species. On average, NEO and NIS species invade the Mediterranean food webs at different levels. The ATL species, being on average dominated by carnivores, invade higher up in the Mediterranean food webs. They probably occupy the empty niches or take advantage of the loose links, resulting from historical and recent fishing-induced removal of top predators (e.g., sharks and seals; see Sala [60]). The opposite is true for NIS. They are, on average, omnivorous and invade lower in the Mediterranean local food webs. Thus, they are embedded at a level allowing them to use the maximum possible trophic links in the Mediterranean food webs (see Sala [60]). This agrees with the results of Harmelin-Vivien et al. [45], who find that most of the Indo-Pacific invaders of the Lebanese rocky coast are herbivores, omnivores, and zooplanktivores (i.e., thus generally having TROPH < 3.5) as opposed to the local fish community. On the contrary, Goren et al. [20] report that there is a trend of RS to invade-up the food web in the eastern Mediterranean, a fact that is not supported by our analyses related to TROPHs as well as the FTGs, which suggests that NIS species are filling in trophic niche gaps at lower trophic levels.

### 4.4. Competition, Invasiveness, and Possible Extirpations

The differential embedding of invaders in the local Mediterranean food webs most probably triggers different ecological processes. Thus, for local species, ATL invaders might be less of a problem in terms of competition when compared to predation. The opposite will be true of Indo-Pacific (NIS) invaders. By invading lower in the food web, when compared to the ATL ones, NIS also take advantage of resources that are more abundant than in higher trophic levels (i.e., because of their closer proximity to primary producers). This allows them to locally build, on average, higher biomasses than the ATL ones, thus reaching commercially exploitable levels. This fact, combined with the higher Q/B (i.e., the number of times a population consumes its own weight in a year (see Pauly [61]) values for RS and NIS might result in additional competition for food resources in the already oligotrophic East Mediterranean. Indeed, existing evidence shows that Indo-Pacific invaders: (i) either competitively displace local species (e.g., [7,44,62]); or (ii) narrow the trophic niche of local species (e.g., Dodecanese Islands: *Siganus luridus* and *S*. *rivulatus* vs. *Boops boops* and possibly *Sarpa salpa* the only other local herbivore: [63]; Libyan waters: *S*. *rivulatus* vs. *B*. *boops*: [63]); and/or (iii) decrease the biomass of local species (e.g., Lebanese waters: *Siganus* spp. vs. *S*. *salpa*: [44]; Israel: *Mullus barbatus* and *M*. *surmuletus* vs. *Upeneus* spp. and *Plotosus lineatus*: [64]).

At present, there is not any evidence suggesting that native species are totally replaced by Indo-Pacific ones, other than reports of displacement and reduced abundances (e.g., [7,10,44,62]). However, competition might lead to extinction and/or extirpation of local species induced by climate change (e.g., [57,58,59]), especially when combined with strong anthropogenic impacts. Fishing, in particular, is one of the most important factors dramatically impacting both coastal [65] and open sea ecosystems [66,67,68] by rapidly diminishing the biomass of the exploited resources and potentially driving species to extinction (e.g., [69]). In the Mediterranean, during the last decades, the intense subsidy-driven modernization of the fishing fleets allowed the expansion of fishing to offshore/deeper grounds, which were not accessible to fishing gears before and thus acted as ‘natural deep refuges’ (e.g., [70,71]). Such an increase in fishing pressure will soon decrease the biomasses of local species to a threshold beyond which competition with NIS might possibly drive some of the local species to extinction. Combined with the low vulnerability and medium-high resilience of NIS species and their low commercial values that make them less prone to fishing, fishing pressure on native species will provide more space for both existing and other potential NIS (i.e., of the >1350 fish species inhabiting the Red Sea species [72] to establish themselves.

### 4.5. Future Research Potential

Undoubtedly this work suffers from certain limitations, with the most debatable being the use of biological traits from the life history key tool of FishBase and not of actual values from the literature. For instance, the present study is based on the use of general TROPHs for all species. However, it is known that TROPHs can vary with time, geographic area, and body size (i.e., ontogenetically) (e.g., [28,29,68]). In addition, TROPHs estimates from diet data are generally associated with some degree of uncertainty [73]. Both these factors might have affected the statistical validity of the mean TROPHs comparisons between the groups. Nevertheless, FishBase is based on literature-reported data, and with the use of elaborate modeling, efforts are made to make as precise estimates of various parameters as possible.

In addition, the present study also leaves many open questions. For example, is it generally true that NEO and NIS invaders primarily occupy open niches? Will there be any future interactions between Atlantic and Indo-Pacific Species, which are at present generally distributed in the western and eastern Mediterranean, respectively? Does the massive influx of Indo-Pacific herbivores further disrupt the benthic ecosystems through overgrazing, and will the omnivores upset the existing degree of omnivory in the Mediterranean food webs? If yes, will this lead to an increase in the predominance of weak species interactions, and what will be the effects of the latter on the stability of the Mediterranean food webs (on a general discussion on this topic, see, e.g., [74])? How will low vulnerability and resilience prove to be of high competitive advantage for Red Sea species invading and establishing in the Mediterranean, especially under the light of overexploitation of stocks? Will there be a shift to fishing métiers toward exploitation of commercial NEO and NIS? Overall, the results presented here could serve as a baseline for future horizon-scanning efforts with respect to potential newcomers in the Mediterranean. Additionally, they provide a rather intriguing hypothesis for future research, which can be based on the use of refined biological trait estimates (e.g., from detailed, area-specific data).

## 5. Conclusions

In general, there was a high degree of overlap in trait variability among the different regions studied here. Based on the analyses performed, it was found that Mediterranean fishes show great affinities in traits with the Atlantic species, whereas differences were observed in all traits with the Red Sea ichthyofauna. Atlantic demersal and pelagic species that prefer high temperatures are most likely to expand their range distribution in the Mediterranean. On the other hand, Red Sea species that are of high resilience and low vulnerability, combined with high longevity (lifespan), are most likely to enter and establish populations in the Mediterranean. Overall, the mean temperature was the strongest predictor of establishment success, followed by habitat type preference, L_inf,_ and L_max_.

The results and analyses presented here could be a useful tool in future horizon-scanning efforts, shedding light on potential newcomers in the Mediterranean. Nevertheless, more refined biological trait information, especially from species-area-specific data, would provide a clearer view of invasion and establishment potential and success.

## Data Availability

The dataset compiled and analyzed within this paper is derived from FishBase, and original data are freely available there. However, the complete dataset used herewithin can be made available upon request.

## Supplementary Materials

The following supporting information can be downloaded at: https://www.mdpi.com/article/10.3390/biology11111625/s1, Table S1: Habitat, trophic groups, resilience, vulnerability, and preferred temperature of Mediterranean inhabitants (MED), Red Sea (RS), Atlantic (ATL), non-indigenous (NIS) and neonative (NEO) species, according to the corresponding fields in FishBase [25]. Trophic groups; H: herbivores; OV: omnivores with a preference for plants; OA: omnivores with a preference for animal material; CD: carnivores with a preference for decapods and fish; CC: carnivores with a preference for fish and cephalopods. Table S2: Biological traits of Mediterranean inhabitants (MED), Red Sea (RS), Atlantic (ATL), non-indigenous (NIS), and neonative (NEO) species, according to the corresponding fields in FishBase [25]. Figure S1: Graphical display of the correlation matrix based on the results of Spearman’s correlation coefficients for each pair of continuous traits after adjusted *p*-values using false discovery rate. Circle areas show the absolute value of corresponding correlation coefficients. X indicates insignificant correlation coefficients. Red color visualizes negative values and blue color positive ones. Mean T: mean preferred temperature; K and L_inf_: von Bertalanffy growth coefficient and infinite length, respectively; L_max_: maximum reported length; L_m_ and t_m_: length and age at maturity, respectively; PD_50_: phylogenetic diversity index; Q/B: food consumption. Figure S2: PCA ordination biplot conducted based on the correlation matrix for 8 traits. Symbols represent the means for each group of species, and trait loadings on the two axes are depicted as vectors. ATL: Atlantic; MED: Mediterranean; NEO: neonatives; NIS: non-indigenous; RS: Red Sea. Mean T: mean preferred temperature; K: von Bertalanffy growth coefficient; L_max_: maximum reported length. Figure S3: Receiver operating characteristic curves for predicting establishment between Red Sea and NIS species; Figure S4: Receiver operating characteristic curves for predicting establishment between Atlantic Sea and NEO species.
