# Supplementary material for: Can Biological Traits Serve as Predictors for Fishes’ Introductions, Establishment, and Interactions? The Mediterranean Sea as a Case Study
Source: Biology (Basel). 2022 Nov 7;11(11):1625. doi: 10.3390/biology11111625 (PMC9687294; doi:10.3390/biology11111625)
Supplement: Supplementary file 1 [file biology-11-01625-s001.zip › biology-1935088-supplementary.pdf]

Table S1. Habitat, trophic groups, resilience, vulnerability, and preferred temperature of Mediterranean inhabitants (MED), Red Sea (RS), Atlantic (ATL), non-indigenous (NIS) and neonative (NEO) species, according to the corresponding fields in FishBase [25]. Trophic groups; H: herbivores; OV: omnivores with a preference for plants; OA: omnivores with a preference for animal material; CD: carnivores with a preference for decapods and fish; CC: carnivores with a preference for fish and cephalopods.

		ATL	NEO	MED	RS	NIS	ATL	NEO	MED	RS	NIS
		Number of species					% number of species				
Habitat	BathyDemersal	72	5	45	5	2	18.8	9.4	8.4	1.0	1.1
	BathyPelagic	194	1	59		1	50.8	1.9	11.0		0.6
	BenthoPelagic	29	10	58	12	9	7.6	18.9	10.8	2.3	5.0
	Demersal	46	24	250	89	42	12.0	45.3	46.5	17.0	23.2
	Pelagic	25	4	78	19	16	6.5	7.5	14.5	3.6	8.8
	Reef-Associated	16	9	48	398	111	4.2	17.0	8.9	76.1	61.3
Trophic groups	H	3	1	3	26	12	0.8	1.9	0.6	5.2	6.7
	OV	5	2	20	45	24	1.3	3.8	3.7	8.9	13.5
	OA	219	19	289	249	87	57.5	35.8	53.9	49.4	48.9
	CD	70	12	99	102	32	18.4	22.6	18.5	20.2	18.0
	CC	84	19	125	82	23	22.0	35.8	23.3	16.3	12.9
Resilience	very low	41	4	49	21	3	11.8	7.7	9.3	4.2	1.7
	low	72	16	94	56	22	20.8	30.8	17.9	11.2	12.4
	medium	151	22	245	169	74	43.6	42.3	46.6	33.7	41.8
	high	82	10	138	255	78	23.7	19.2	26.2	50.9	44.1
Vulnerability	10–19	72	5	94	171	46	18.8	9.4	17.5	33.1	25.4
	20–29	72	8	94	120	47	18.8	15.1	17.5	23.2	26.0
	30–39	73	6	107	85	38	19.1	11.3	19.9	16.4	21.0
	40–49	69	14	61	57	16	18.1	26.4	11.3	11.0	8.8
	50–59	41	6	55	35	18	10.7	11.3	10.2	6.8	9.9
	60–69	28	11	61	21	9	7.3	20.8	11.3	4.1	5.0
	70–79	12	2	36	16	6	3.1	3.8	6.7	3.1	3.3
	80–89	13	1	28	11		3.4	1.9	5.2	2.1	
	90–100	2		2	1	1	0.5		0.4	0.2	0.6
Mean preferred temperature (°C)	2–4	60		7			16.9		1.4		
	4–6	61		13			17.1		2.6		
	6–8	52	4	18	1	1	14.6	7.7	3.6	0.2	0.6
	8–10	47	3	38		2	13.2	5.8	7.5		1.2
	10–12	43	7	82			12.1	13.5	16.2		
	12–14	20	4	65	1	2	5.6	7.7	12.9	0.2	1.2
	14–16	19	2	75	3	1	5.3	3.8	14.9	0.6	0.6
	16–18	9	1	38	2	2	2.5	1.9	7.5	0.4	1.2
	18–20	7	9	99	3		2.0	17.3	19.6	0.6	
	20–22	9	2	10	4	2	2.5	3.8	2.0	0.8	1.2
	22–24	4	1	16	5	8	1.1	1.9	3.2	1.0	4.8
	24–26	11	5	17	7	17	3.1	9.6	3.4	1.5	10.2
	26–28	14	14	27	255	76	3.9	26.9	5.3	53.1	45.5
	28–30				199	56				41.5	33.5
Preferred temperature range (°C)	0–2	32	2	18	4	4	8.9	3.8	3.5	0.8	2.4
	2–4	61	5	59	86	20	17.0	9.6	11.6	18.0	11.8
	4–6	79	12	89	320	93	22.0	23.1	17.5	66.8	55.0
	6–8	55	10	80	36	26	15.3	19.2	15.7	7.5	15.4

	8–10	61	13	90	15	16	17.0	25.0	17.7	3.1	9.5
	10–12	33	5	85	12	6	9.2	9.6	16.7	2.5	3.6
	12–14	20	1	36	4	2	5.6	1.9	7.1	0.8	1.2
	14–16	6	3	23	1	1	1.7	5.8	4.5	0.2	0.6
	16–18	8		22	1		2.2		4.3	0.2	
	>18	4	1	6		1	1.1	1.9	1.2		0.6

Table S2. Biological traits of Mediterranean inhabitants (MED), Red Sea (RS), Atlantic (ATL), non-indigenous (NIS) and neonative (NEO) species, according to the corresponding fields in FishBase [25].

		ATL	NEO	MED	RS	NIS	ATL	NEO	MED	RS	NIS
		Number of species					% number of species				
Life span (longevity; years)	0–1	3		4	14	2	1.0		0.8	2.8	1.2
	1–2	13		29	58	4	4.2		5.8	11.7	2.4
	2–3	16		34	54	21	5.1		6.7	10.9	12.4
	3–4	32	3	41	46	19	10.3	5.8	8.1	9.3	11.2
	4–5	20	4	35	43	20	6.4	7.7	6.9	8.7	11.8
	5–6	20	3	25	42	16	6.4	5.8	5.0	8.5	9.4
	6–7	23	3	37	26	11	7.4	5.8	7.3	5.3	6.5
	7–8	19	2	22	18	12	6.1	3.8	4.4	3.6	7.1
	8–9	16	3	24	20	7	5.1	5.8	4.8	4.0	4.1
	9–10	10	2	21	22	5	3.2	3.8	4.2	4.4	2.9
	10–20	79	17	132	108	37	25.4	32.7	26.2	21.8	21.8
	20–30	30	10	53	25	13	9.6	19.2	10.5	5.1	7.6
	30–40	16	2	21	11	1	5.1	3.8	4.2	2.2	0.6
	40–50	9	1	18	4	2	2.9	1.9	3.6	0.8	1.2
	50–60	2	1	1	1		0.6	1.9	0.2	0.2	
	70–80	2	1	6	2		0.6	1.9	1.2	0.4	
	90–100			1	1				0.2	0.2	
	> 100	1					0.3				
Generation time (years)	0–1	26	1	37	92	21	8.3	1.9	7.3	18.6	12.4
	1–2	63	8	120	157	62	20.2	15.4	23.8	31.7	36.5
	2–3	72	10	96	86	31	23.1	19.2	19.0	17.4	18.2
	3–4	39	8	61	48	18	12.5	15.4	12.1	9.7	10.6
	4–5	32	4	57	45	11	10.3	7.7	11.3	9.1	6.5
	5–6	15	4	18	17	12	4.8	7.7	3.6	3.4	7.1
	6–7	12	5	19	15	9	3.8	9.6	3.8	3.0	5.3
	7–8	13	1	23	8	1	4.2	1.9	4.6	1.6	0.6
	8–9	5	2	14	2		1.6	3.8	2.8	0.4	0.0
	9–10	6	4	13	6	1	1.9	7.7	2.6	1.2	0.6
	>10	29	5	46	19	4	9.3	9.6	9.1	3.8	2.4
Age at maturity (t_m; years)	0–1	34	1	75	134	38	10.9	1.9	14.9	27.1	22.4
	1–2	99	13	142	154	65	31.7	25.0	28.2	31.1	38.2
	2–3	58	12	100	91	23	18.6	23.1	19.9	18.4	13.5
	3–4	45	8	66	54	24	14.4	15.4	13.1	10.9	14.1
	4–5	20	7	34	29	9	6.4	13.5	6.8	5.9	5.3
	5–6	8	2	29	9	6	2.6	3.8	5.8	1.8	3.5
	6–7	16	4	16	10	3	5.1	7.7	3.2	2.0	1.8
	7–8	15		15	5		4.8		3.0	1.0	
	8–9	5	2	12	4		1.6	3.8	2.4	0.8	
	9–10	4	1	6		2	1.3	1.9	1.2		1.2
	> 10	8	2	8	5		2.6	3.8	1.6	1.0	
Length at maturity (L_m; cm)	0–5	42		64	78	5	11.2		12.0	15.1	2.8
	5–10	61	2	80	102	36	16.3	3.8	15.0	19.7	20.1
	10–15	59	6	71	71	29	15.7	11.3	13.3	13.7	16.2
	15–20	54	6	54	61	31	14.4	11.3	10.1	11.8	17.3
	20–25	32	8	38	48	19	8.5	15.1	7.1	9.3	10.6

	25–30	31	2	36	35	14	8.3	3.8	6.7	6.8	7.8
	30–35	24	8	28	27	8	6.4	15.1	5.2	5.2	4.5
	35–40	11	3	17	13	8	2.9	5.7	3.2	2.5	4.5
	40–45	4	5	20	19	6	1.1	9.4	3.7	3.7	3.4
	45–50	12	6	9	11	3	3.2	11.3	1.7	2.1	1.7
	50–55	12	3	16	14	5	3.2	5.7	3.0	2.7	2.8
	55–60	2	1	14	4	3	0.5	1.9	2.6	0.8	1.7
	60–65	3	1	9	7	6	0.8	1.9	1.7	1.4	3.4
	65–70	6		5	2	1	1.6		0.9	0.4	0.6
	70–75	4		8	1		1.1		1.5	0.2	
	75–80			8	5				1.5	1.0	
	80–85	3	1	3	2		0.8	1.9	0.6	0.4	
	85–90			7	1	1			1.3	0.2	0.6
	90–95			7		1			1.3		0.6
	95–100	5	1	5	1	2	1.3	1.9	0.9	0.2	1.1
	> 100	10		35	15	1	2.7		6.6	2.9	0.6
Infinite length (L_{inf}; cm)	0–10	70		93	108	17	18.3		17.4	20.9	9.5
	10–20	63	3	88	109	38	16.5	5.7	16.4	21.1	21.2
	20–30	62	8	59	65	29	16.2	15.1	11.0	12.6	16.2
	30–40	42	7	46	50	29	11.0	13.2	8.6	9.7	16.2
	40–50	30	5	38	47	16	7.9	9.4	7.1	9.1	8.9
	50–60	27	6	33	25	9	7.1	11.3	6.2	4.8	5.0
	60–70	20	4	24	27	6	5.2	7.5	4.5	5.2	3.4
	70–80	6	3	19	14	9	1.6	5.7	3.5	2.7	5.0
	80–90	11	4	14	15	4	2.9	7.5	2.6	2.9	2.2
	90–100	8	7	5	8	3	2.1	13.2	0.9	1.5	1.7
	100–110	10	3	21	13	4	2.6	5.7	3.9	2.5	2.2
	110–120	1		9	2	2	0.3		1.7	0.4	1.1
	120–130	3	1	8	8	6	0.8	1.9	1.5	1.5	3.4
	130–140	6		5	1	1	1.6		0.9	0.2	0.6
	140–150	4		8	1		1.0		1.5	0.2	
	150–160			6	5				1.1	1.0	
	160–170	3	1	4	2	1	0.8	1.9	0.7	0.4	0.6
	170–180			4	1	1			0.7	0.2	0.6
	180–190			8		1			1.5		0.6
	190–200			2	1				0.4	0.2	
	> 200	16	1	42	15	3	4.2	1.9	7.8	2.9	1.7
K (1/years)	0–0.1	30	5	47	19	3	9.6	9.6	9.3	3.8	1.8
	0.1–0.2	54	18	96	69	30	17.3	34.6	19.0	13.9	17.6
	0.2–0.3	59	9	96	69	21	18.9	17.3	19.0	13.9	12.4
	0.3–0.4	33	7	57	49	19	10.6	13.5	11.3	9.9	11.2
	0.4–0.5	35	4	48	36	19	11.2	7.7	9.5	7.3	11.2
	0.5–0.6	21	2	29	46	14	6.7	3.8	5.7	9.3	8.2
	0.6–0.7	17	3	22	31	15	5.4	5.8	4.4	6.3	8.8
	0.7–0.8	16	3	17	27	8	5.1	5.8	3.4	5.5	4.7
	0.8–0.9	12		15	14	10	3.8		3.0	2.8	5.9
	0.9–1	6	1	17	13	6	1.9	1.9	3.4	2.6	3.5
	1–1.1	3		14	17	3	1.0		2.8	3.4	1.8
	1.1–1.2	4		5	15	6	1.3		1.0	3.0	3.5
	1.2–1.3	4		5	10	7	1.3		1.0	2.0	4.1
	1.3–1.4	1		6	5	2	0.3		1.2	1.0	1.2

	1.4–1.5	3		2	10	1	1.0		0.4	2.0	0.6
	1.5–2	7		18	27	2	2.2		3.6	5.5	1.2
	2–2.5	1		3	15	2	0.3		0.6	3.0	1.2
	2.5–3	3		5	11		1.0		1.0	2.2	
	3–3.5				5					1.0	
	3.5–4	1		1	3	1	0.3		0.2	0.6	0.6
	4–4.5				2					0.4	
	4.5–5	1				1	0.3				0.6
	> 5	1		2	2		0.3		0.4	0.4	
Food consumption (Q/B)	0–5	143	25	215	55	23	37.4	47.2	40.0	10.7	12.9
	5–10	123	16	155	134	59	32.2	30.2	28.8	26.0	33.1
	10–15	53	5	62	92	28	13.9	9.4	11.5	17.8	15.7
	15–20	20	6	34	51	14	5.2	11.3	6.3	9.9	7.9
	20–25	12	1	8	24	10	3.1	1.9	1.5	4.7	5.6
	25–30	9		8	22	10	2.4		1.5	4.3	5.6
	30–35	2		11	19	13	0.5		2.0	3.7	7.3
	35–40	4		10	19	6	1.0		1.9	3.7	3.4
	40–45	3		3	14	3	0.8		0.6	2.7	1.7
	45–50	2		12	10	4	0.5		2.2	1.9	2.2
	50–55			4	9	4			0.7	1.7	2.2
	55–60	1		3	6		0.3		0.6	1.2	
	60–65	5		3	7		1.3		0.6	1.4	
	65–70			2	3	2	0.0		0.4	0.6	1.1
	70–75	1		2	13	1	0.3		0.4	2.5	0.6
	75–80	1		1	5	1	0.3		0.2	1.0	0.6
	80–85				2					0.4	
	85–90	1			9		0.3			1.7	
	90–95				4					0.8	
	95–100			1	2				0.2	0.4	
	> 100	2		4	16		0.5		0.7	3.1	

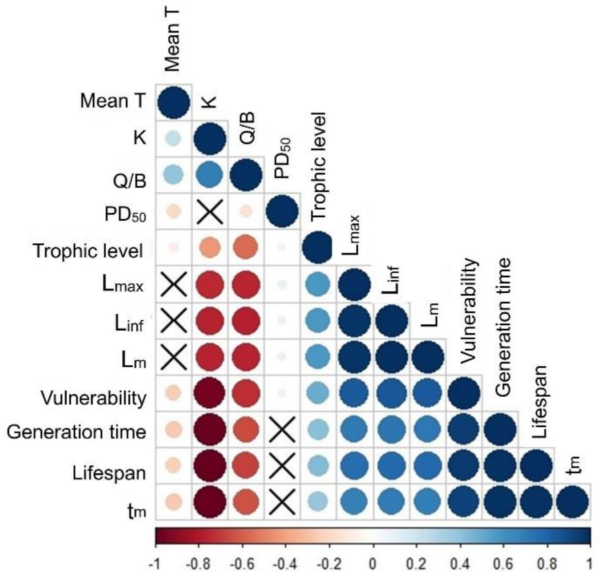

Figure S1. Graphical display of the correlation matrix based on the results of Spearman's correlation coefficients for each pair of continuous traits, after adjusted p values using false discovery rate. Circle areas show the absolute value of corresponding correlation coefficients. X indicates insignificant correlation coefficients. Red colour visualizes negative values and blue colour positive ones. mean T: mean preferred temperature; K and L_{inf}: von Bertalanffy growth coefficient and infinite length, respectively; L_{max}: maximum reported length; L_m and t_m: length and age at maturity, respectively; PD_{50}: phylogenetic diversity index; Q/B: food consumption.

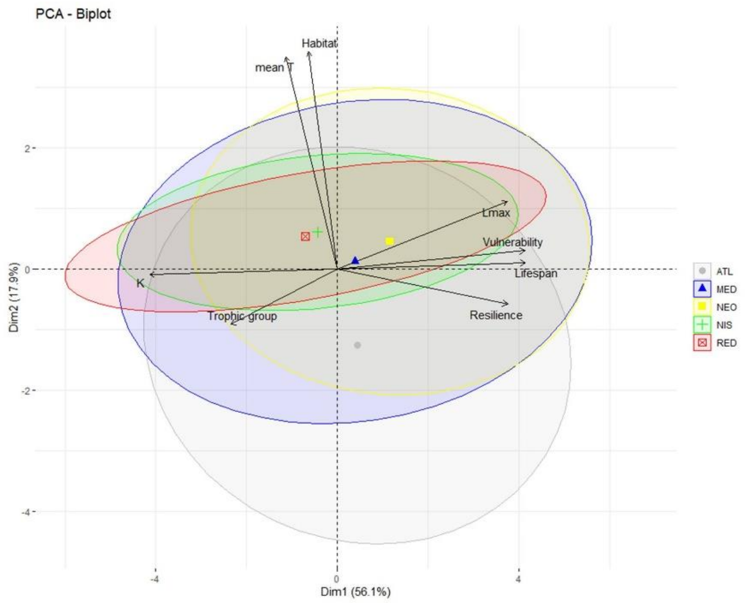

Figure S2. PCA ordination biplot conducted based on the correlation matrix for 8 traits. Symbols represent the means for each group of species and trait loadings on the two axes are depicted as vectors. ATL: Atlantic; MED: Mediterranean; NEO: neonatives; NIS: non-indigenous, RS: Red Sea. mean T: mean preferred temperature; K: von Bertalanffy growth coefficient; Lmax: maximum reported length.

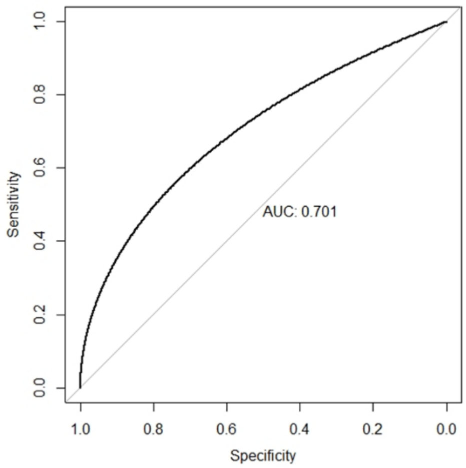

Figure S3. Receiver operating characteristic curves for predicting establishment between Red Sea (RS) and non-indigenous (NIS) species.

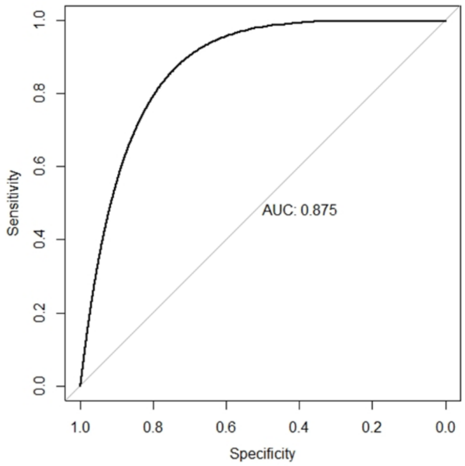

Figure S4. Receiver operating characteristic curves for predicting establishment between Atlantic Sea (ATL) and neonative (NEO) species.
